# Effect of 1110 MBq Radioiodine in Reducing Thyroid Volume in Multinodular Goiter: A New Protocol

**DOI:** 10.4021/jocmr1361w

**Published:** 2013-04-23

**Authors:** Armando Flores-Rebollar, Aida Ruiz-Juvera, Guadalupe Lopez-Carrasco, Ofelia Gonzalez-Trevino

**Affiliations:** a Department of Internal Medicine, Instituto Nacional de Ciencias Medicas y Nutricion “Salvador Zubira”, DF, Mexico; b Department of Nuclear Medicine, Instituto Nacional de Ciencias Medicas y Nutricion “Salvador Zubira”, DF, Mexico

**Keywords:** Methimazole, Radioiodine, Multinodular goiter, Thyroid, Uptake, ^131^I, Treatment, Hyperthyroidism

## Abstract

**Background:**

There is no consensus on the optimal treatment of multinodular goiter (MNG), but in the past few years, the use of radioiodine has increased. This study’s objective was to evaluate adjuvant methimazole (MMI) therapy to increase and standardize radioiodine uptake (RAIU) with a fixed therapeutic ^131^I dose of 1110 MBq (30 mCi).

**Methods:**

Our study included 5 women with MNG treated with MMI, 10 - 15 mg/day for 2 to 4 months, prior to the administration of 1110 MBq ^131^I (30 mCi); none of the patients developed hypothyroidism during MMI therapy and had average basal TSH levels of 0.32 ± 0.39 mIU/L that increased to 2.6 ± 0.9 mIU/L (P = 0.07).

**Results:**

RAIU increased from 25.6 ± 8.7% to 49.2 ± 8.3% (P = 0.003). All patients were followed for 12 months: median thyroid volume (TV) decreased from 77.2 mL (32.9 - 124.2) to 48.8 ml (12.4 - 68.9) with an average decrease of 46.4 ± 14.8% (P = 0.01). All patients developed hypothyroidism during the first 6 months after radioiodine therapy.

**Conclusions:**

This new therapeutic protocol using MMI as adjuvant therapy is effective in increasing RAIU as well as the deleterious effects of ^131^I, without increasing the required dose, but leading to thyroid volume decreases similar to those reported with the use of recombinant human thyrotropin (rhTSH) or higher radioiodine doses.

## Introduction

Worldwide, multinodular goiter (MNG) is a common thyroid pathological entity, frequently associated to a deficient iodine intake and other factors [1]. Clinical manifestations hinge on glandular growth, compression and functional autonomy.

No consensus has been reached in terms of MNG treatment and its optimal management remains controversial [2]; therapeutic options include levothyroxine (LT4), surgery and radioiodine. Suppressive treatment with LT4 is discouraged due to the possible risk of causing sub-clinical or overt hyperthyroidism as well as its low efficacy when compared with other treatment modalities [3]. Total or sub-total thyroidectomy effectively decrease thyroid volume and its related complications, but carry the risk of anesthetic and surgical complications [2]. The use of ^131^I in the treatment of MNG has increased in the past few years as a result of its use in hyperthyroidism for over 70 years [4]. In some European and Latin American countries, ^131^I therapy replaced surgery as the treatment of choice in patients with MNG [5]. This change was a result of several factors: many patients prefer non-surgical treatment, ^131^I therapy can be conducted on an ambulatory basis and cost-benefit analysis favors radioiodine. MNG volume reduction depends on the basal thyroid volume, the amount of administered ^131^I and the gland’s uptake capacity. Published reports on the use of ^131^I have referred thyroid volume reductions of 35 to 60% [4, 6]. The use of ^131^I is limited by low and heterogeneous radioiodine uptake (RAIU), particularly in regions or countries with normal diets or those with high iodine intake. A low RAIU leads to a less effective treatment and renders this treatment modality more difficult [4]. Low RAIU can be overcome with the use of recombinant human thyrotropin (rhTSH) but it has only been approved for well-differentiated thyroid cancer; regardless in the past decade, its use as coadjuvant MNG therapy in association with ^131^I, in order to increase RAIU and obtain a homogeneous ^131^I distribution, has become more frequent [7-12]. The obtained results have been comparable to the use of ^131^I but with the advantages of lower dosage and the possibility of ambulatory therapy.

Treatment with rhTSH is expensive and in some countries, the price is prohibitive. A Brazilian group [13] recently reported that increasing endogenous TSH after methimazole (MMI) therapy increased RAIU from 21 to 78% and decreased thyroid volume by 46% after ^131^I administration; TSH was increased to an average of 11.7 ± 5.4% mIU/L. Similar results have been reported by others [14].

The purpose of this study is to attempt this new RAIU increase protocol with methimazole and endogenous TSH, in a group of patients with MNG treated with a fixed ^131^I dose of 1110 MBq (30 mCi), evaluate the results and compare them with those reported in the literature.

## Materials and Methods

### Patients

Five (5) MNG patients were selected from the Internal Medicine Outpatient Clinic of the “Instituto Nacional de Ciencias Medicas y Nutricion” “Salvador Zubiran”. All were newly admitted patients for the management of their thyroid problem and in search of an alternative to surgical treatment (thyroidectomy); they had never been treated with MMI, thyroid hormones or radioactive iodine. None had associated severe comorbidities or heart disease. The study followed the Institute’s Bioethics Committee’s guidelines and all patients signed a consent form.

Basal thyroid volume (TV) was determined by ultrasound in all cases and malignancy was ruled out by ultrasound-guided fine needle biopsy of suspicious nodules. Serum TSH, free T4 (FT4), total T3 (TT3), anti-thyroid peroxidase antibodies (TPOAb) and anti-thyroglobulin antibodies (TgAb) were determined before the thyroid scan and basal ^131^I measurement. Three patients had sub-clinical hyperthyroidism and two were euthyroid; all were treated with MMI, 10 - 15 mg/day during their first visit and after the previously mentioned evaluation. Subsequent visits were scheduled every 4 weeks at which time TSH, FT4 and TT3 levels were again determined. We attempted to maintain FT4 above 9.0 pmol/L and avoid hypothyroidism; the degree of clinical hypothyroidism was estimated using the score developed by Zulewski et al [15] (Hypothyroid, more than 5 points; euthyroid less than 3 points, intermediate 3 - 5 points); MMI dosage was decreased as necessary. The goal was to increase by 50% the basal RAIU without conditioning clinical hypothyroidism; once reached, 1110 MBq (30 mCi) ^131^I were administered orally and the patients were re-evaluated monthly for the first 4 months and later, every 2 months for a year. Clinical signs of dysthyroidism, thyroid volume by US, TSH, FT4, TT3, TPOAb and TgAb were determined at each visit.

### Methods

A thyroid scan with 1.85 MBq (50 μCi) ^131^I and 24 hour uptake determination were conducted in all patients (Gammacamara, Model E-Cam, Siemens). Thyroid ultrasound was performed with portable General Electric (GE) equipment and a 60 mm lineal transducer (7.5 MHz). Nodules with suspicious characteristics were biopsied under ultrasound guidance. Thyroid volume was determined by ultrasound and the volume of each thyroid lobe was calculated with the ellipsoid volume equation (0.52 × width × height × length). Total thyroid volume was obtained by adding the volume of both lobes.

Serum TSH, FT4, TT3, TPOAb and TgAb concentrations were measured by radioimmunoassay. FT4 had an analytical sensitivity of 0.65 pmol/L (normal range: 9.0 - 23.2 pmol/L), the TT3 assay had an analytical sensitivity of 0.15 nmol/L (normal range: 0.9 - 2.9 nmol/L), TgAb had an analytical sensitivity of 2.0 IU/mL (normal values: under 30 IU/mL (RIA-gnost^®^ FT4, RIA-gnost^®^ T3, TGAB ONE STEP^®^, Cisbio Bioassays, France). The assay used in TPOAb determination had an analytical sensitivity of 1.9 IU/mL (normal values: under 100 IU/mL); TSH was determined by immunoradiometric assay (IRMA) and had an analytical sensitivity of 0.005 mIU/L (normal range: 0.3 to 4.0 mIU/L) (Anti-hTPO RIA KIT^®^, Turbo TSH ^125^I IRMA KIT^®^ Izotop Budapest, Hungary).

### Statistical analysis

Statistical analysis was conducted with the SPSS 15.0 program (Chicago IL, USA). Results are presented as medians (ranges) or mean ± SD and depending on the data’s distribution, Kolmogorov-Smirnov’s test.

Differences over time were analyzed with Student’s paired t test. Statistical significance was established at P < 0.05.

## Results

All patients were female. One year after radioiodine administration, the median TV decreased from 77.2 mL (32.9 - 124.2) to 48.8 ml (12.4 - 68.9). The average TV decrease was 46.4 ± 14.8% (P = 0.010) (Table 1).

**Table 1:** Basal Patient Characteristics, After MMI Pretreatment and One Year After Radioiodine

Patient	Age (years)	Basal TSH (mUI/L)	Time on MMI (months)	Dose of MMI (mg)	RAIU Pre-MMI (%)	RAIU Post-MMI (%)	TSH at ^131^I* (mUI/L)	Basal TV (mL)	Post-^131^I TV (mL)†	TV Reduction (%)
1	60	0.02	4	15	17	50	2.99	77.2	51.7	33.0
2	56	0.40	2	10	31	45	0.98	70.6	48.8	30.9
3	58	0.07	3	10	20	38	0.74	124.2	68.9	45.2
4	75	0.15	2	10	22	53	1.10	32.9	12.4	62.4
5	63	0.96	2	10	38	60	6.0	82.5	32.6	60.5
Mean	62.4	0.32	2.6	11.0	25.6	49.2	2.36	77.5	42.9	46.4
SD	7.5	0.39	0.9	2.2	8.7	8.3	2.22	32.6	21.4	14.8
Median								77.2	48.8	
Range								32.9 - 124.2	12.4 - 68.9	

*Before radioiodine administration and 4 days after MM withdrawal; †Measured 12 months after radioiodine; MMI: Methimazole; RAIU: Radioactive iodine uptake; TV: Thyroid volume.

The RAIU increase due to MMI pre-treatment went from 25.6 ± 8.7% to 49.2 ± 8.3% (P = 0.003). The effective ^131^I released dose per gram of thyroid tissue was 8.47 ± 5.53 MBq (229.0 ± 149.6 μCi).The initial MMI dose in all patients was 10 mg/day except in one case that initially received 15 mg/day. Three (3) patients reached the RAIU goal within 2 months, and the others, by the third and fourth month of therapy; ^131^I was administered on the fourth day after MMI withdrawal. Our patients did not tolerate the initial MMI doses for more than 4 weeks since they all reached the lower FT4 limits and 2 patients developed mild hypothyroidism symptoms, (Zulewski’score) [15]. Average basal TSH was 0.32 ± 0.39 mIU/L and increased to 2.6 ± 0.9 mIU/L (P = 0.07) after the brief exposure to MMI.

After ^131^I administration, only one patient developed severe radiation-related thyroiditis (case 5) that required steroid therapy for a week. None of the patients presented an increase in anti-thyroid antibodies, except for one case whose TPOAb increased seven months after ^131^I administration but with no associated hyperthyroidism or clinical findings suggestive of Grave’s disease.

All patients developed hypothyroidism after radioactive iodine within the first 6 months after therapy. Clinical hypothyroidism was diagnosed when Zulewski’s score was above 5 points in association with a FT4 level < 9.0 pmol/L and a TSH level > 4.0 mIU/L. Thyroid hormone substitution therapy was initiated with per os sodium levothyroxine; the dose was calculated on a 1.6 μg/kg/day basis, administering a total of 25 μg/day that was progressively increased until TSH was steadily maintained between 1.0 and 3.0 mIU/L and hypothyroidism symptoms resolved.

## Discussion

Although this study included a small number of patients, it showed that pre-treatment with MMI increases radioiodine uptake and renders it homogeneous without compromising its efficacy; it increased its deleterious effects and hence, decreased TV. In contrast with the previous study [13], we did not determine a target TSH level; the purpose was to reach a 50% or above increase in the basal RAIU after MMI treatment. This was achieved with minimal MMI doses, still maintaining TSH within normal limits and avoiding clinical hypothyroidism and observing symptomatic improvement. The decreases observed in our study were similar to those in the previously referred study (46.4 vs. 46.2%) [13] and when compared with studies that used a fixed dose of 1110 MBq (30 mCi) ^131^I, our results are similar or superior to ^131^I with or without rhTSH pretreatment [9, 16, 17]. The mechanism by which the thyroid RAIU increased can only be explained by TSH-mediated activation of the sodium-iodine symporter (NIS) [18]. Although the TSH increase was not significant, the tendency is clear but perhaps blunted by the small number of patients. Although the reported data in the literature may differ in terms of pre-treatment with anti-thyroid drugs and the effectiveness of radioiodine in benign thyroid disease, this study and the experience in Brazil have shown the benefits of pre-treatment, particularly in the improvement of thyroid RAIU. Perhaps, the moment of MMI withdrawal at the time of ^131^I administration, under 7 days in both studies, may influence these positive results [18, 19]. Surprisingly, all our patients developed hypothyroidism after therapy; this could be explained by increased radioiodine sensitivity in our patients or to the fortuitous aggregation of individuals with radiosensitivity per se. However, it has been shown that individuals pre-treated with MMI and undergoing ^131^I therapy for MNG have a higher incidence of hypothyroidism than those that are not pre-treated with MMI [6].

As in the Brazilian study [13], we decided to use a fixed radioiodine dose, the maximum dosage was allowed by Mexican guidelines in ambulatory treatment; moreover, most patients had large goiters and low RAIU. The difference hinged on establishing thyroid uptake as the treatment goal and therefore avoiding the possibility of developing clinical hypothyroidism. The risk of presenting side effects due to MMI was averted by using low doses and this may have also prevented an increase in TV by substantially increasing TSH levels. The average time period required to reach this uptake goal was 2.6 ± 0.9 months, an appropriate time period considering the delays in radioiodine treatments within an institutional medicine context in our country.

Although the new American Thyroid Association (ATA)/American Association of Clinical Endocrinologists (AACE) [20] guidelines recommend ^131^I as the therapy of choice in MNG, they do not support the use of rhTSH as an adjuvant to radioiodine therapy; it is not clear, however, whether pretreatment with MMI in toxic MNG is recommendable. They do refer in guideline number 35, that the Brazilian study obtained similar results to the use of rhTSH with MMI pretreatment.

Our study is small, heterogeneous and only includes female patients but we have proven as in the South American studies, that this therapeutic option effectively decreases TV in MNG. Future studies should focus on homogeneous groups and compare the effects of this alternative with the use of rhTSH in the treatment of MNG.

## Declaration of Interest

The authors had no conflicts of interest to declare in relation to this article.

## Author Contribution

Armando Flores-Rebollar: Concept/design, Data analysis/Interpretation, Statistics/data collection, Drafting article and Critical revision of article; Aida Ruiz-Juvera: Data analysis/Interpretation, Data collection, Drafting article and Critical revision of article; Guadalupe Lopez-Carrasco: Data analysis/Interpretation, Data collection and Critical revision of article; Ofelia Gonzalez-Trevino: Data analysis/Interpretation, Data collection and Critical revision of article.
